# Caregiving for Older Adults With Dementia During the Time of COVID-19: A Multi-State Exploratory Qualitative Study

**DOI:** 10.1177/07334648231175414

**Published:** 2023-05-26

**Authors:** Kevin Yan, Tonie Sadler, Daniel Brauner, Harold A. Pollack, R. Tamara Konetzka

**Affiliations:** 1 Perelman School of Medicine, 14640 University of Pennsylvania, Philadelphia, PA, USA; 2 Shirley Ryan AbilityLab, Feinberg School of Medicine, Institute for Public Health and Medicine, 12244 Northwestern University, Chicago, IL, USA; 3 Departments of Medicine, Family and Community Medicine and Medical Ethics, Humanities and Law, Division of Geriatrics, Homer Stryker M.D. School of Medicine, 51374 Western Michigan University, Kalamazoo, MI, USA; 4 Crown Family School of Social Work, Policy, and Practice, Public Health Sciences, Urban Health Lab, 278762 University of Chicago, Chicago, IL, USA; 5 Department of Medicine, Section of Geriatrics and Palliative Medicine, Public Health Sciences, 2462 University of Chicago, Chicago, IL, USA

**Keywords:** Alzheimer's disease, caregiving, COVID-19, home- and community-based care and services, nursing homes, long-term services and supports

## Abstract

This qualitative semi-structured interview study explores how 64 family caregivers for older adults with Alzheimer’s Disease and related dementias across eight states experienced and executed caregiving decisions before and during the COVID-19 pandemic. First, caregivers experienced challenges communicating with loved ones and healthcare workers in all care settings. Second, caregivers displayed resilient coping strategies in adapting to pandemic restrictions, finding novel strategies to balance risks while preserving communication, oversight, and safety. Third, many caregivers modified care arrangements, with some avoiding and others embracing institutional care. Finally, caregivers reflected on the benefits and challenges of pandemic-related innovations. Certain policy changes reduced caregiver burden and could improve care access if made permanent. Telemedicine’s increasing use highlights the need for reliable internet access and accommodations for individuals with cognitive deficits. Public policies must pay greater attention to challenges faced by family caregivers, whose labor is both essential and undervalued.

**What this paper adds**

• We explore caregivers’ lived experiences and perceptions of policies that impacted their pandemic-era decision-making across states varying in HCBS utilization.
• We describe ways that communication challenges and difficulties assessing care quality eroded caregivers’ trust in nursing homes and HCBS during the pandemic.
• We present great variability in caregivers’ pandemic-era decisions, with some caregivers reducing formal care, while others newly opted for nursing homes or HCBS to lighten increasing care burdens.

**Applications of study findings**

• Our findings inform efforts to make a more resilient and equitable health system through policy changes to improve supports for caregivers, a critical support population for older adults with dementia.
• We describe policy changes and technological innovations that expanded care access during the pandemic and that could improve health equity if made permanent.
• Caregivers’ observations regarding telemedicine’s increasing role underscore the public health imperative of reliable and high-quality internet access in all communities.

## Introduction

Older adults with Alzheimer’s Disease and Related Dementias (ADRD) face particular risks of COVID-19 morbidity and mortality (Xia et al., 2021). These risks arise partially because ADRD causes overexpression of pro-inflammatory molecules and reduced ability to care for oneself, increasing COVID-19 morbidity. Moreover, congregate settings like nursing homes increase COVID-19 transmission risk (Konetzka et al., 2021; Xia et al., 2021). By August 2022, U.S. long-term care (LTC) facility residents represented less than 0.5% of the U.S. population and fewer than 3% of U.S. COVID-19 cases, yet accounted for more than 15% of U.S. COVID-19 deaths (CDC, 2022; CDC, 2023). Non-institutional settings experienced similar challenges; most states experienced home- and community-based services (HCBS) workforce shortages and the permanent closure of at least one Medicaid HCBS provider (Watts et al., 2021). To counteract COVID-19, U.S. authorities implemented COVID-19 transmission reduction measures, including social distancing, personal protective equipment (PPE) mandates, and facility capacity restrictions. LTC facilities took additional measures, including limiting visitors and decreasing social activities (Dobbs et al., 2020; Lightfoot & Moone, 2020; Simard & Volicer, 2020).

LTC facility visitation bans complicate caregivers’ care quality monitoring. Decreased physical and social activities can compound cognitive and physical decline and increase mortality (Simard & Volicer, 2020). Furthermore, COVID-19 exacerbated caregiver burden by worsening access to healthcare providers and causing loss of usual caregiving supports (White et al., 2022). Pandemic-related economic stresses like unemployment also threatened caregivers’ ability to provide care (Falk et al., 2021). Pandemic-related uncertainty increased caregiver responsibilities, while COVID-19 health risks heightened caregiver stress and burden (Masoud et al., 2022).

Literature examining COVID-19’s impact on older adults with ADRD has focused on older adults’ health outcomes and how pandemic-response measures affected caregivers in specific care settings and geographic areas (Brown et al., 2020; Lai et al., 2020; Miller, 2020; Tousi, 2020). Few studies have rigorously explored caregivers’ lived experiences and perceptions of policies that impacted their pandemic-era decision-making and wellbeing across different care settings and states that vary in HCBS utilization.

Our study addresses this gap, examining how family caregivers for older adults with ADRD in eight states experienced and made care decisions across nursing homes and HCBS as COVID-19 began. We employ Anderson’s expanded Behavioral Model of Health Services Use to better understand how caregivers navigated, accessed, and made decisions about LTC in the COVID-19 context (Anderson, 1995; Anderson & Newman, 2005). We confirm prior studies’ findings that demonstrate telemedicine can improve care access, especially for ADRD caregivers, and we identify potential implementation barriers (O'Connor et al., 2023). We conducted qualitative, semi-structured interviews with caregivers about caregiving relationships, factors underlying decision-making between HCBS and institutional care, family outcomes, and COVID-19’s impact on these experiences.

Our paper contributes to long-term services and supports (LTSS) and caregiving research in three main ways. First, we provide specific examples of how older adults with ADRD and their caregivers were impacted by COVID-19 in institutional and home-based settings, especially during the pandemic’s turbulent early months. Second, we document caregiver challenges and successes that illuminate how agencies can improve adaptability, communication, and continuing pandemic response. Last, we provide recommendations for how the healthcare community can re-examine LTSS and caregiving in light of lessons learned from the COVID-19 pandemic.

## Methodology

### Sample Recruitment

We used purposive, quota sampling to recruit 59 family caregivers for people over age 65 living with ADRD, plus five family caregivers for people over age 60 living with ADRD with experiences relevant to the research questions, for semi-structured, in-depth interviews (Table 1). We recruited participants from Arkansas, Florida, Illinois, Minnesota, New York, North Carolina, Oregon, and Texas. States were selected for variation in HCBS percentage of Medicaid LTSS expenditures, from Florida’s 33% to Oregon’s 81% (Eiken et al., 2018). Participants were limited to caregivers who self-reported making important care decisions within 12 months of interview screening, such as choosing HCBS.

**Table 1.** Demographic Characteristics of Care Recipients.

Demographic Characteristic	Overall, *N* = 67^1^
State, *n* (%)	
Illinois	18 (27%)
Florida	9 (13%)
New York	8 (12%)
Oregon	8 (12%)
Texas	8 (12%)
Minnesota	7 (10.5%)
Arkansas	5 (7.5%)
North Carolina	4 (6%)
Sex, *n* (%)	
Male	43 (64%)
Female	24 (36%)
Race, *n* (%)	
White	52 (78%)
Black or African American	8 (12%)
Asian	3 (4%)
American Indian or Alaska Native	1 (1.5%)
More than one race	1 (1.5%)
Unspecified	2 (3%)
Ethnicity, *n* (%)	
Hispanic	2 (3%)
Non-Hispanic	65 (97%)
Age	
Mean (range)	78 (61–97)
Primary payer source, *n* (%)	
Medicare	33 (49%)
Medicaid-Medicare	24 (36%)
Veterans Affairs insurance	7 (10.5%)
Medicaid only	3 (4.5%)

1 Of the 64 study participants, three reported caring for two individuals. Their respective demographic information is included in this table.

Recruitment primarily involved Facebook advertising to obtain a diverse sample from rural and urban communities in eight states (Corey et al., 2018). Ads targeted individuals with identified interest in “Family caregivers,” “Caregiver,” “Alzheimer’s Association,” or “Home Care Giver,” and linked to the Facebook page, “The Caregiver Survey,” containing contact and study information, including funding source and participant compensation. $4362 spent on Facebook advertisements produced 35,824 clicks or pageviews. Participants were also recruited from caregiving support groups, state and national caregiver advocacy groups, and LTC facilities and hospitals networked with a university medical center. Interested individuals were screened for eligibility and guided through verbal informed consent before full-length interviews were scheduled. A $75 gift card was offered for participation in full-length interviews, and a $25 gift card was offered for participation in follow-up interviews. This study received approval by University of Chicago BSD IRB17-0865 and was funded by a research grant from the National Institute on Aging (NIA RF1 AG054071).

### Interview Protocol Development

The interview guide was generated by an interdisciplinary team of six medical, public health, and social work researchers. This team conducted informational interviews with six leaders in ADRD health research and policy to shape initial protocol development. Pilot interviews were conducted with six Illinois caregivers. Following pilot interviews, the research team modified interview and recruitment protocols to improve data quality, including narrowing questions to four specific areas: (1) background underlying the caregiving relationship, (2) events, considerations, and people impacting decision-making between HCBS and institutional care, (3) effect of state policy, social services, insurance, and finances on decision-making and caregiver burden, and (4) patient and family outcomes. A final semi-structured interview protocol was completed prior to data collection (Appendix A). After the COVID-19 pandemic began, we added questions to the final interview protocol and created a follow-up interview protocol to investigate COVID-19’s impact on the wellbeing of older adults with ADRD and their caregivers and caregiver decision-making (Appendix B).

### Conducting Interviews

Two authors (KY and TS) conducted 69 telephone interviews, including five follow-up interviews, with 64 unique caregivers between April 2019 and February 2021. We conducted 21 full-length interviews before and 43 after the COVID-19 pandemic began, totaling 64 caregivers. We contacted the 21 participants who interviewed before the pandemic to participate in follow-up interviews about their COVID-19 experiences. Five of these completed a follow-up interview, bringing the total number of interviews to 69. The remaining 15 participants did not respond or refused the follow-up interview. Full-length interviews typically lasted one to 2 hours and follow-up interviews lasted approximately 30 minutes.

Caregiver recruitment continued until thematic saturation, defined as the absence of new themes in four categories: 1) caregiver decision-making, 2) policy impact on decision-making, 3) caregiver decision outcomes, and 4) COVID-19’s impact on decision-making.

### Data Analysis

All 69 transcribed interviews, including the 21 interviews conducted before the COVID-19 pandemic, were used in the analysis of caregiving experiences prior to and during the COVID-19 pandemic. Interviews were voice recorded, professionally transcribed, and coded using NVivo 12 software for qualitative and mixed methods research (QSR International, 2020). We wrote analytic memos for each interview, summarizing interview content and details about general observations and participant mood. We used thematic and content analysis methods to create codes based on study research questions and interview themes (Creswell & Poth, 2017). Codebook development involved an iterative process with a) initial codes based on protocol topics, b) code adjustments and new theme additions as interviews were conducted, and c) code contextualization using thematic and narrative summaries after all interviews were completed. Using the finalized codebook, two authors, KY and TS, coded each interview independently, meeting regularly to reconcile coding disagreements and to edit the codebook until consensus was achieved (Appendix C).

The research team reviewed the coded interviews, narrative summaries, and interview memos to explore caregiver experiences related to decision-making and LTC services. To better understand caregivers’ COVID-19-related experiences, we extracted four coding nodes and their associated subnodes for focused analysis of the post-COVID interviews: (1) COVID-19 and caregiver experiences, (2) COVID-19 and formal care, (3) COVID-19 and state policy, (4) COVID-19 and decision-making. We used thematic and content analysis methods to organize and report patterns in the data, extract meaningful statements, and formulate themes present (Creswell & Poth, 2017; Vaismoradi et al., 2013). The research team discussed and reconciled conflicting opinions on theme contents.

## Results

Our analysis revealed four major COVID-19-related themes (Table 2).

**Table 2.** Study Themes and Topics.

Themes, Sub-topics	Example Quotations
Communication	
Assessing care	*No one cared. It was a nightmare. I kept calling different departments, trying to get somebody to help me and look in on her…That’s how COVID affected us…I was not allowed in, and the doctor tried to make an exception. There were no exceptions, they would not allow me in the hospital, and there was no one to advocate for my mom*
	*COVID is convenient now to use as a coverup…[T]hey can do whatever the hell they want to these people, and we don’t know about it because we’re not allowed in the facilities*
Clinic closures	*When COVID hit, [the clinic] shut down. And they’ve got four or five offices scattered around the region here; they all closed up. Come to find out…nobody was able to see a doctor for a while. Couldn’t get in–There was nobody there to answer the phone, give an appointment, or nothing*
Routine and adaptability	
Dementia-related challenges	*[I]t’s kind of been hard because he has dementia, and he doesn’t understand why he can’t go outside…COVID hit all of us; then he was locked down. And now he’s really confused because he remembers routine, but he can’t remember, like day to day, that there’s a reason why he can’t leave the facility and why I can’t come in to see him.*
	*She’s very social. And staying social is very important to keeping the cognitive decline from progressing faster. When she started getting trapped in the house…there’s nothing for her to do except sit and just stare at the wall*
Disrupted routines	*It’s been really hard. I mean, I work from home. I work full-time. I have kids that are in school. My kids are doing homeschool…I’m trying to be a caregiver for her. I don’t have any relief, really, aside from my husband, who’s also working. So, it’s very hard…We had utilized home health care as needed, but we made the decision not to do that during this time because of the possible cross-contamination and so forth for our whole family*
	*So, I’d see them all the time. And when my folks moved into the assisted living, I would see them…probably four days a week. And now with COVID-19, I can’t go in the building to see her. But I try almost every day to go visit her* via *the balcony. Obviously, I can’t go inside. So, anything I need to get her, I have to drop it off at the desk, and they will take it up to her*
Care decisions	
Nursing home	*I’ve seen on the news where they have some family members locked in those facilities and they can’t even see their family members…I’m not willing to submit my family member to where they can be abused. So I just ain’t doing that, not at all*
	*Ever since the whole COVID-19, it’s almost pretty much gone off the table. [I] don’t trust having him sent to a place. I’d much rather have him stay with family at home…*
	*…[a]nd I’m so glad he was there when this hit because I don’t know what I would’ve done if I’d had to have people come in and out of my house*
Home- and community-based services (HCBS)	*When she started getting trapped in the house… sometimes she’ll read a book, but otherwise there is nothing for her to do. Absolutely nothing for her to do, except to stare at the walls. So we decided we were going to get somebody in to be with her*
	*I just don’t like letting strangers into my home…I probably wouldn’t have one of them come in, definitely while COVID*
Remote healthcare	
Telemedicine	*I actually love it, because I don’t have to be in the waiting room and just sitting there waiting. It’s quicker virtual*
	*I actually like it. I mean, he has no risk [of] going out, being infected. Because he does have asthma and other breathing issues, so I try to be extra careful with that*
	*I have to go twice a year for my doctor just to keep my prescriptions going. We tried to renew online over the telemedicine. My internet connection wouldn’t allow for that kind of a visit. We have internet, but it’s like 3.5. We’re supposed to be getting a lot more, but we get very poor signal*
Other remote interventions	*[T]hey deliver our medicine now. We don’t have to go get it. Now they deliver it to us. Our doctor’s appointments, he only got to come in if there’s something serious. I got a temperature thing, so I take his temperature here at home, [to] make sure he’s good. He ain’t been coughing, he ain’t got no issues, but if he does, I call them and let them know. That’s what they want me to do, they want me to call them and let them know, that way they can say if I can come in or not*
	*Yes, and that was to do with sending this box home that connects…it’s through your phone line, so that they can get his blood sugar and blood pressure reading immediately. And I mean, as soon as he does it…I can get a phone call before he’s even up from the chair if there’s something off. So I mean, they are right on top of it…So that was a policy change that they had, I think partly due to the Coronavirus…It’s been a blessing for us*
	*Actually, it’s gotten easier because the government agencies have cut down on bureaucracy and paperwork. And a lot of them are not even open for in-person, so there’s not much bureaucracy…Everybody’s on the same page for a change, instead of having to get the same information several different times*

First, caregivers reported significant challenges communicating with loved ones and healthcare workers.

Second, caregivers displayed resilient coping strategies to disruptions to routine.

Third, some caregivers modified care arrangements and the trajectory of future care arrangements.

Finally, caregivers reflected on the benefits and challenges of telemedicine and pandemic-induced technological policy innovations.

### Theme 1: Communication Challenges

Difficulties communicating with healthcare facilities, abrupt service terminations, and uncertainties about COVID-19 infection left caregivers feeling isolated. Caregivers cited inability to assess care, quality and safety, challenges to participation in medical appointments, and inability to communicate consistently with loved ones. The pandemic further burdened caregivers through reduced or lost service supports.

Allison^6^ is the primary caregiver for her 94-year-old mother living in Florida. Her mother was hospitalized after experiencing overnight health complications. Allison described how communication difficulties and uncompromising visitation limits during her mother’s hospitalization obstructed her care involvement and advocacy:

I kept calling, "How are you treating her if no one knows her background?" No one cared. It was a nightmare. I kept calling different departments, trying to get somebody to help…That's how COVID affected us…I was not allowed in…There were no exceptions…There was no one to advocate for my mom.

Allison’s mother was non-verbal and stopped eating while hospitalized. Worried that her mother might not survive long, Allison sought to bring her home. To do that, Allison was required to place her mother on hospice, though she did not believe her mother needed end-of-life care.

Similarly, Bridget, another caregiver, recounted communication difficulties that impeded her care involvement and weakened her trust in her mother’s nursing home. She described:

COVID is convenient now to use as a coverup…They can do whatever the hell they want to these people, and we don't know about it because we're not allowed in the facilities. Before, it was happening and they were making excuses…Now, they can say whatever they want because nobody's going in to check…I have to call around and get answers from doctors because I'm not getting the information from the home…even though I'm the legal guardian.

To caregivers like Allison and Bridget, pandemic-era communication challenges increased distrust by obstructing caregivers’ ability to monitor nursing home services and to advocate for loved ones. Bridget, already skeptical of nursing home care quality, was now unable to personally ensure her mother was safe.

Leah, caregiver for her 75-year-old cousin in Arkansas, abruptly lost contact with her cousin’s primary care clinic once COVID-19 began. She explained:

When COVID hit, [the clinic] shut down…They've got four or five offices scattered around the region…they all closed…Nobody was able to see a doctor for a while. Couldn't get in–There was nobody there to answer the phone, give an appointment, or nothing.

Caregivers like Leah suffered repercussions of facility closures occurring without notice at the pandemic’s onset, hindering services access.

In home-based settings, COVID-19 infection risk and pandemic policy responses exacerbated existing staffing and services access problems. Jackie, caregiver for her 94-year-old mother in Illinois, described how her HCBS workers made understandable decisions to quit working, worsening her care burden:

With the COVID, we had homemakers that didn’t want to come because they were getting unemployment, and happy to get unemployment. And then I think they think that we are possible carriers of COVID, and they don’t want to be exposed.

Across care settings, caregivers believed that the pandemic hampered their abilities to access services and advocate for loved ones. Caregivers expressed frustration about worsening communication and oversight over medical institutions, weakening institutional trust.

### Theme 2: Disruptions of Routine and Adaptability

Within nursing home and HCBS settings, routines are indispensable to people with ADRD and their caregivers. Many caregivers maintained routine communication with family members through video calls and outdoor visits. Maintaining routines was complicated by pandemic policy fluctuations. Moreover, dementia-related memory loss can hinder one’s ability to understand and comply with mask-wearing and other health restrictions (Brauner et al., 2000).

Leah, the Arkansas caregiver noted above, explained how her cousin’s dementia impeded his understanding of pandemic-related changes in a nursing home:

[It’s] hard because he has dementia and he doesn't understand why he can't go outside…COVID hit…then he was locked down. And now he's really confused because he remembers routine, but he can't remember, like day to day, that there's a reason why he can't leave the facility and why I can't come in to see him.

Outside LTC facilities, some caregivers canceled HCBS and related supports to mitigate infection risk. Lauren, caring for her 68-year-old mother in Florida, made this decision despite working full-time while homeschooling children. She revealed:

It's been really hard…I work from home. I work full-time…My kids are doing homeschool…I'm trying to be a caregiver for her. I don't have any relief, really, aside from my husband, who's also working. So, it's very hard…We had utilized home healthcare as needed, but we made the decision not to do that during this time because of the possible cross-contamination and so forth for our whole family.

For caregivers like Lauren, COVID-19 forced a choice between shouldering greater caregiving workloads and placing family members at greater infection risk.

Caregivers also voiced concerns about isolation and its exacerbation of cognitive decline. They recounted distancing-related isolation at home and decreased activities, group events, and visitation in institutional settings. Janet, caregiver for her 62-year-old mother in Florida, equated social distancing with being “trapped” at home:

She's very social. And staying social is very important to keeping the cognitive decline from progressing faster. When she started getting trapped in the house…there's nothing for her to do except sit and just stare at the wall.

Jeremy, caregiver for his 61-year-old father in New York, even compared nursing home isolation to imprisonment:

The government is doing what they're doing right now, which I understand, but my heart wouldn't feel the same. Like, what do you mean I can't see my dad? I'm not about to have that. I guess you should call the police and lock me up too, since you're locking him up…

While caregivers like Janet and Jeremy understand the value of restrictive pandemic-mitigating policies, isolation is burdensome to caregivers and care recipients.

Despite greater physical and emotional burdens, caregivers adapted to pandemic-related restrictions by using electronic communication**,** and finding socially distanced ways to meet loved ones. Amee, caregiver for her 90-year-old mother in Florida, described how she maintained frequent contact despite pandemic distancing policies by adapting her visitations:

[W]ith COVID-19, I can't go in the building to see her. But I try almost every day to go visit her via the balcony…Anything I need to get her, I have to drop it off at the desk, and they will take it up to her.

Caregivers like Amee endeavored to minimize pandemic disruptions by finding new ways to maintain care routines, demonstrating the premium caregivers place on face-to-face interactions. Routine disruptions distressed older adults with ADRD and their caregivers, but caregivers adapted to preserve care practices and care frequency. Caregivers assumed additional responsibilities during COVID-19 as usual caregiving supports were lost.

### Theme 3: Changes in Care Setting and Future Care Decisions

The pandemic had varied effects on families’ care utilization and care setting choices. As COVID-19 deaths rose among older adults, many caregivers reduced their formal care use. Still others sought new HCBS as COVID-19 increased caregiving burdens or appreciated nursing facilities that were communicative and that quickly instituted protective measures.

New York caregiver Jeremy, introduced in Theme 2, described how news reports contributed to his decision to absolutely refuse nursing home care:

I've seen on the news where they have some family members locked in those facilities and they can't even see their family members…I'm not willing to submit my family member to where they can be abused. So I just ain't doing that, not at all.

Jeremy saw nursing home visitation restrictions as abuse. Shawn, caregiver for his 66-year-old father in Florida, also commented that developments as COVID-19 progressed undermined his trust in LTC facilities:

[S]ince the whole COVID-19, it’s almost pretty much gone off the table. [I] don’t trust having him sent to a place.

For caregivers like Jeremy and Shawn, harrowing stories about the fate of nursing home residents during the pandemic led them to refuse consideration of nursing homes.

In contrast, other caregivers reported positive experiences with the cleanliness and professionalism of nursing homes. Dana, caregiver for her 85-year-old mother in Texas, noted:

The last few years of my mother's life have all just been so excellent. The cleanliness and the new construction and the professional and cheerful demeanor of everybody, administrators, as well as staff on the floor…I try not to watch the news, but of course you see the stories and the numbers even if you try to avoid them. And what I see on TV or read on the internet does not jibe with my experience. My experience with those kinds of facilities has been very positive.

Similarly, Leah, from Arkansas, appreciated that her cousin lived in a LTC facility because HCBS would entail people frequenting her own home, posing COVID-19 exposure risks. She remarked:

I'm so glad he was there when this hit because I don't know what I would've done if I'd had to have people come in and out of my house.

Thus, caregivers like Dana and Leah preferred LTC facilities to the risk of bringing COVID-19 into the home and appreciated quality care provided by facility staff.

In the home-based setting, Janet from Florida described how she initiated HCBS to mitigate her mother’s pandemic-related socialization decline:

When she started getting trapped in the house…there is nothing for her to do…So we decided we were going to get somebody in to be with her.

However, Amee, caregiver for her 82-year-old mother in Oregon, remarked that COVID-19 infection risk exacerbated her skepticism toward high-turnover HCBS staff. Evaluating the potential acquisition of a home health aide, she mentioned:

I just don't like letting strangers into my home…I probably wouldn't have one of them come in, definitely [not] while COVID-19 is going around.

Overall, caregivers reacted differently to pandemic-related burdens, changing service utilization based on personal circumstance and risk assessment. Caregiver distrust of LTC facilities increased, but some caregivers were relieved that their loved ones were living in communicative, well-managed nursing facilities. Similarly, while some caregivers increased HCBS utilization as care burdens intensified, others saw COVID-19 as reason to terminate services or decline additional HCBS.

### Theme 4: Innovations and Telemedicine

From caregivers’ experiences, certain policy innovations decreased caregiver burden: waiving Medicare’s 3-day inpatient hospitalization requirement for skilled nursing facility coverage, medication delivery, at-home vitals monitoring, and reduced in-person requirements for healthcare services qualification. Caregivers also reported that telemedicine enabled more-responsive, less-burdensome, and safer family-provider communication, although cognitive impairment and poor internet service hampered some older adults' telemedicine use.

Judy, caregiver for her 87-year-old father in North Carolina, praised telemedicine’s efficiency:

I actually love it, because I don’t have to be in the waiting room and just sitting there waiting. It’s quicker, virtual.

Kate, caregiver for her 75-year-old father in Oregon, appreciated telemedicine reducing infection risk:

I actually like it. I mean, he has no risk [of] going out, being infected…He does have asthma and other breathing issues. So I try to be extra careful with that.

Some medical institutions lessened barriers to remotely receive medications, monitor health, and communicate with health professionals. This increased healthcare’s accessibility and decreased infection risks. Jeremy, the caregiver from New York, lauded the expanded access to doctors, medication, and health monitoring:

They deliver our medicine now. We don't have to go get it. Now they deliver it to us. Our doctor's appointments, he only got to come in if there's something serious. I got a temperature thing, so I take his temperature here at home, make sure he's good…He ain't got no issues, but if he does, I call them and let them know.

Skylar, caregiver for her 72-year-old husband in Texas, similarly extolled innovations that facilitated real-time family-provider communication about vitals:

[They sent] this box home that connects…it's through your phone line, so that they can get his blood sugar and blood pressure reading immediately…As soon as he does it…I can get a phone call before he's even up from the chair if there's something off…They are right on top of it…So that was a policy change that they had, I think partly due to the Coronavirus…It's been a blessing for us.

Caregivers like Jeremy and Skylar reaped the benefits of more-responsive, convenient, and accessible care innovations spurred by COVID-19.

Government agencies’ actions and Medicare policy changes designed to limit time spent in congregate settings sometimes also facilitated improved services access.

Gerard, for example, explained how COVID-era changes reduced bureaucracy and inter-agency confusion, making obtaining services easier for his 84-year-old mother in North Carolina:

Actually, it's gotten easier because the government agencies have cut down on bureaucracy and paperwork. And a lot of them are not even open for in-person, so there's not much bureaucracy…Everybody's on the same page for a change, instead of having to get the same information several different times.

Amee, from Florida, similarly appreciated how COVID-related Medicare policy changes regarding subacute rehabilitation improved her health system experience**:**

It’s a 100-day benefit period for Medicare, once you have a 3-day qualifying hospital stay. But they’re not making you have the 3-day qualifying hospital anymore, because of COVID.

Although telehealth was convenient and improved accessibility for caregivers, technological complexities challenged individuals with ADRD. Kristen, caregiver for her 65-year-old husband in North Carolina, recounted telehealth technology upsetting her husband, who preferred phone communication**:**

They actually gave him a tablet; which like I said, he doesn't even have a big computer. He tried the tablet, and he got upset and put it back in the box and sent it back…and said he doesn't want it…He wants to do it on the phone.

Inadequate internet service further impeded access to telehealth and other health services in underserved urban and rural areas. Floyd, caregiver for his 84-year-old mother in rural Arkansas, explained that poor internet precluded him from utilizing telemedicine:

I have to go twice a year for my doctor just to keep my prescriptions going. We tried to renew online over the telemedicine. My internet connection wouldn't allow for that kind of a visit. We have internet…but we get very poor signal. We can't watch Netflix or nothing on our internet.

For caregivers like Floyd, structural impediments like subpar internet access may exacerbate health disparities and create care access difficulties as telehealth expands.

## Discussion

Our interviews identified many challenges facing caregivers in the time of COVID-19. Communication difficulties with HCBS and institutional facilities undermined caregivers’ care access and oversight, eroded trust in medical facilities, and altered care decision-making. Our findings corroborate literature showing the pandemic worsened caregiver burden through loss of usual caregiving supports and interference with care quality monitoring (Simard & Volicer, 2020; White et al., 2022). Policy fluctuations and routine disruptions further complicated caregivers’ formal care oversight, making caregivers assume additional responsibilities, with greater risk of adverse health outcomes (Masoud et al., 2022).

Participants’ varied responses also underscored the diversity of caregivers’ COVID-19 experiences in nursing home and HCBS settings. COVID-19 mortality in nursing homes has been well documented, but our report of positive experiences with institutional care and caregiver concerns with HCBS demonstrate that HCBS are not viable alternatives for all caregivers to remedy institutional care deficits. Lastly, our research supports literature showing telemedicine reduces care access barriers, especially for ADRD caregivers for whom caregiving responsibilities and travel constraints impose heightened difficulties (O'Connor et al., 2023).

### Implications for Policy, Practice, and Research

Individuals and institutions scrambled to respond in myriad ways to novel challenges and varied family situations during the COVID-19 pandemic. Our healthcare system must adapt to better protect older adults with ADRD and their families by facilitating provider-family communication, minimizing disruptions to routine activities, and reducing health service access barriers.

Our study underscores the importance of implementing LTC provider guidelines that facilitate communication between families and staff during emergencies, and that reduce policy and service fluctuations to minimize routine disruptions. Such guidelines could also promote mechanisms for regular audiovisual contact between residents and family.

Corroborating prior studies, our findings demonstrate the value of institutional linkages with existing community connections like Area Agencies on Aging to maintain continuity of care and to coordinate consistent responses across the health system (Miller et al., 2020; Wasserman et al., 2022). Strict nursing home enforcement of infection-mitigation measures reduced quality of life for residents and family. In conjunction with health regulators, facilities should establish formal guidelines for in-person visitation alternatives, discontinued social activity replacements, and means whereby families can participate in doctors' appointments and be informed of ongoing care conditions.

We also identified a breakdown in caregivers’ trust in institutional facilities and HCBS connected to COVID-19. Many caregivers suspected that nursing homes were places of rampant infection and abuse. Moreover, the comings-and-goings of HCBS staff, who frequently displayed high turnover, posed infection risks for care recipients and other household members.

To recover confidence in health services safety, specific interventions are needed to support the wellbeing of family caregivers and LTC recipients in health emergencies. Policies ensuring a living wage, sick leave, and health insurance for LTC workers could reduce healthcare workforce infections, mitigating transmission risk to care recipients, and improve care quality through reduced turnover and sufficient staffing (NASEM, 2022). Paying family caregivers may also lessen pressure on caregivers to rely upon institutional care, or to risk infection to afford caregiving assistance in home settings.

Our interviews indicate that permanently implementing pandemic-related innovations and procedural hurdle reductions could improve care access and quality. Remote medication delivery and health monitoring could make medicine more equitable and accessible while reducing infection risks. Reduced bureaucracy, shorter in-person wait times, and fewer requirements for obtaining health services are particularly beneficial for caregivers. Permanently waiving the 3-day hospitalization requirement to qualify for subacute rehabilitation for persons with ADRD who could clearly benefit from subacute rehabilitation, but less so from hospitalization, should be considered.

Our results also underscore the importance of continuing and expanding telemedicine. Caregivers appreciate telehealth’s accessibility and safety improvements (O'Connor et al., 2023). As telemedicine becomes an enduring and essential part of the healthcare system, internet access will become a public health issue. Our interviews showcase how individuals residing in underserved urban and rural areas can encounter difficulties utilizing telemedicine**. A**ssistance is needed to establish reliable and high-quality internet access for all people.

Lastly, our findings suggest important areas for continuing research. Future studies should investigate how perspectives and relative spending on HCBS, compared to institutional care, have changed in light of the COVID-19 pandemic. The instance of an older patient with ADRD having to enroll in hospice to leave the hospital was concerning, and the extent of prioritization of liability considerations above patient welfare should be examined. Moreover, our interviews illustrate great variation in caregiver response to institutional policy changes, with some caregivers even increasing service utilization. Researchers and policymakers should study the actions of facilities that received praise from families. Such positive examples can inform facility-caregiver communication and pandemic-response practices moving forward.

### Strengths and Limitations

This study’s strengths include its focus on caregiver perspectives around healthcare decision-making for family members with dementia during the height of COVID-19’s impact. We used a rigorous qualitative research design involving in-depth, open-ended interviews with 64 unique caregivers across eight states varying by HCBS expenditure rates. Using thematic analysis, we identified and interpreted four themes with important impact on caregiver decision-making.

While we broadly sampled across states, our sample size was relatively small and may be unrepresentative of caregivers generally, which limited our ability to draw cross-state policy comparisons that may impact caregiver decision-making.

## Conclusion

The COVID-19 experience revealed severe deficiencies in our LTC system. Communication challenges between family caregivers and providers, loss of usual caregiving supports, and policy disruptions that undermined normal routines increased caregiver burden and eroded caregivers’ trust in health services.

Decisions about care settings were nuanced and family-specific, with no clear or uniform best choices. Formal guidelines for caregiver-provider communication might improve caregiver trust. Moreover, policymakers should make permanent pandemic-related policy changes that reduce bureaucratic obstacles, like relaxing requirements for obtaining health services such as location requirements for telemedicine and hospital stay requirements for subacute rehabilitation. Future research should identify and disseminate institutional best-practices in these domains. As we enter a new-normal COVID-19 era, we have the obligation and opportunity to apply lessons learned to transform LTSS and to create a more accessible, equitable, and resilient healthcare system.

## Supplemental Material

Supplemental Material - Caregiving for Older Adults With Dementia During the Time of COVID-19: A Multi-State Exploratory Qualitative Study Click here for additional data file. Supplemental Material for Caregiving for Older Adults With Dementia During the Time of COVID-19: A Multi-State Exploratory Qualitative Study by Kevin Yan, Tonie Sadler, Daniel Brauner, Harold A. Pollack, R. Tamara Konetzka in Journal of Social and Personal Relationships.

Supplemental Material - Caregiving for Older Adults With Dementia During the Time of COVID-19: A Multi-State Exploratory Qualitative Study Click here for additional data file. Supplemental Material for Caregiving for Older Adults With Dementia During the Time of COVID-19: A Multi-State Exploratory Qualitative Study by Kevin Yan, Tonie Sadler, Daniel Brauner, Harold A. Pollack, R. Tamara Konetzka in Journal of Social and Personal Relationships.

Supplemental Material - Caregiving for Older Adults With Dementia During the Time of COVID-19: A Multi-State Exploratory Qualitative Study Click here for additional data file. Supplemental Material for Caregiving for Older Adults With Dementia During the Time of COVID-19: A Multi-State Exploratory Qualitative Study by Kevin Yan, Tonie Sadler, Daniel Brauner, Harold A. Pollack, R. Tamara Konetzka in Journal of Social and Personal Relationships.
