# Supplementary material for: Caregiving for Older Adults With Dementia During the Time of COVID-19: A Multi-State Exploratory Qualitative Study
Source: J Appl Gerontol. 2023 May 26;42(10):2078–88. doi: 10.1177/07334648231175414 (PMC10214026; doi:10.1177/07334648231175414)
Supplement: Supplemental Material - Caregiving for Older Adults With Dementia During the Time of COVID-19: A Multi-State Exploratory Qualitative Study [file sj-pdf-1-jag-10.1177_07334648231175414.pdf]

Appendix A: Protocol for Semi-Structured Interviews with Caregivers

Background

1. Please tell me about your relationship with X.
2. How long have you been involved in the care of X?
3. Can you take me through a typical day as a caregiver?
4. Are there times of the year when it becomes more difficult to care for X?

Topic 1: How do caregivers think about the choice between HCBS and nursing home care?

Was there a tipping point when you or other caregivers realized you needed more services to help care for X?

1. What did you do when you realized that X needed more care?
2. What were the care options available for you when you were making decisions about X's care? Who presented these options?
3. Did you feel like you had a choice in the type of long-term care services X received?
4. Do you have any previous experiences in your family or among close friends that influenced what kind of care services you wanted for the person you care for?
5. Who helped you make the decision of which long term care services to use?
   - What role did X play in the decision-making process? What role are they playing now in these decisions?
   - Was there a person who was the main decision-maker besides yourself? Why that person?
   - Were there differences in opinion, and how did you resolve those differences?
   - Did information from a physician help with your care decisions?
   - Did information from a care coordinator or social worker help with your care decisions?
   - Did information from a website help with your care decisions?
6. What were the main reasons you or other people involved in the care of X chose [HCBS/nursing home care]?
   - If experienced with both: How would you describe the benefits and drawbacks of both HCBS and nursing home care?
7. Did you consider [whichever option they did not select]? What were the main reasons you decided not to pursue that option?
8. Was your decision influenced by your family members' jobs or schedules, and their availability to be at-home caregivers?
9. How was your decision effected by concerns about physical safety and accessibility in the living environment of the person you care for?
10. Did you modify the home of the person you care for at any time?
11. How did you evaluate the quality of services in making your decisions?
12. What role did the physical demands of caregiving play in the decisions you made?
13. Do you belong to any organization (ex. Facebook group) where you talk to other people in similar situations?

Topic 2: How does state policy impact the choice between HCBS and nursing home care? Do available options and the decision-making process differ between states with different Medicaid policies?

1. Has X moved or changed residency since he/she began receiving care?
2. How would you evaluate the care X has received at each location he/she has lived in?
3. Do you have a case manager or social worker whose job it is to help you manage X's care?

   If yes:

   a) How did you get connected with your case manager/social worker?
   b) When was the first time you talked to them?
   c) When was the last time you talked to them?
   d) What do they do that is helpful for X?
   e) What would you like them to be doing that they aren't?

   If no:

   a) Have you ever thought about retaining services from a case manager/ social worker? Why or why not?
   b) What services were you told X is eligible for?
   c) Were you ever told X was ineligible for a service? Why was X ineligible?
   d) Were you ever told that you would have to wait for a service, and how long?
   e) Did anyone suggest you should pursue Medicaid to get services your family needs?
4. Did you ever try to sign X up for Medicaid?

   If yes:

   a) What happened? How was that experience?
   b) Did someone help you sign up for Medicaid? Who was that?
   c) Has receiving Medicaid been helpful for X and/or X? How so?
   d) Have you and/or X experienced challenges with receiving Medicaid (ex. Redetermination)?

   If no:

   a) Is there a reason that you have not tried to sign X up for Medicaid?
   b) Does X have another form of insurance? Please describe the insurance or other community care plan.
   c) Did someone help you sign up for this other form of insurance?
   d) Has receiving this insurance been helpful for X and/or X? How so?
   e) Have you and/or X experienced challenges with receiving this insurance?

We understand it can be challenging to talk about finances, but we would like to ask a few questions about the topic because it is often an important consideration for caregivers. I would also like to remind you that everything you report during this interview will remain confidential.

1. What aspects of caregiving have been the greatest financial burden?
2. How do you pay for X's care that is not covered by insurance?
3. Do other family/friends assist with care or contribute financially?
4. Does X contribute financially to the household?
5. Are there other expenses related to the family that have been made more difficult due to the cost of X's care?

Topic 3: What have been the outcomes of the decision for the patient and family?

1. What service is most useful for your own life?
2. What would you like to see more of in terms of services?
3. Do you foresee anything you might need to change about the current long-term care arrangements for X? Do you think you'll be able to do this? Why or why not?
4. Given what you have experienced, is there any advice you would offer to other families that are in similar situations to yours?
5. What do you think are the main challenges of receiving care in a [nursing home/HCBS] setting?
6. What do you think are the main successes of receiving care in a [nursing home/HCBS] setting?
7. How has your role as a caregiver changed since your loved one [moved to a nursing home/received HCBS]?
8. What is it like to be a caregiver with a family member in [nursing home care/HCBS]?
9. What has surprised you about this process?
10. If you could go back and make the process easier, what would you change, or where would you have needed help?
11. Do you wish you had made a different decision?

Close-out

Thank participant for participating; emphasize how helpful s/he's been. Make sure s/he knows how to contact you if he has any questions or concerns.

1. Do you know anyone else who may be interested in participating in this study?
2. Is there anything else you would like to add about your caregiving experiences?
