# Supplementary material for: Caregiving for Older Adults With Dementia During the Time of COVID-19: A Multi-State Exploratory Qualitative Study
Source: J Appl Gerontol. 2023 May 26;42(10):2078–88. doi: 10.1177/07334648231175414 (PMC10214026; doi:10.1177/07334648231175414)
Supplement: Supplemental Material - Caregiving for Older Adults With Dementia During the Time of COVID-19: A Multi-State Exploratory Qualitative Study [file sj-pdf-2-jag-10.1177_07334648231175414.pdf]

Appendix B: Protocol for Semi-Structured Interviews with Caregivers: COVID-19 Supplement

Protocol for Semi-Structured _FOLLOW-UP_ Interviews with Caregivers

POST-ONSET OF COVID-19 IN UNITED STATES

Introduction

Thank you for agreeing to take part in this brief interview. As we have discussed, this is a follow-up interview for those who took part in this research study prior to the onset of COVID-19 pandemic in the United States. Due to the significant impact that the coronavirus has had on individuals, families, and communities with healthcare needs, it is important that we understand the extent of the impact COVID-19 has had on those suffering from Alzheimer's and other dementias and their caregivers.

1. Let's start by briefly revisiting your relationship with Person X. For the recording, can you please reiterate whom it is that you care for, the nature of your relationship and how long you have been involved in the care of X? (*Note: Confirm whether X receives in-home care or nursing home care. Also confirm whether X receives Medicaid HCBS or services through private insurance.*)
2. Our initial interview took place on [date of initial interview]. What sorts of changes in caregiving have you experienced since that time?
    - Which, if any, of these changes are specific to the onset of the COVID-19 pandemic?

Topic 1: How do caregivers think about the choice between HCBS and nursing home care?

1. Has the COVID-19 pandemic changed the way you think about decision-making when it comes to your caregiving?
    - Has the coronavirus prompted you to consider services through Medicaid and/or nursing home care that you had not previously considered?
    - Has COVID-19 affected how you think about nursing home placement versus at home care?

Topic 2: How does state policy impact the choice between HCBS and nursing home care? Do available options and the decision-making process differ between states with different Medicaid policies?

1. Has the COVID-19 pandemic impacted your ability to access services through HCBS or nursing home care?
2. Who helps you to consider options and make decisions regarding your caregiving of X?
3. Have health care and social service workers such as a physician, care coordinator or social worker played a role in decision-making since the onset of the COVID-19 pandemic?

We understand it can be challenging to talk about finances, but we would like to ask a few questions about the topic because it is often an important consideration for caregivers. I would also like to remind you that everything you report during this interview will remain confidential.

1. Since the beginning of the spread of the coronavirus in the U.S.:

- What aspects of caregiving have introduced new financial burdens?
- Have any previous financial burdens become lighter following changes to Medicaid, unemployment benefits and/or government stimulus checks received as a result of the coronavirus?

Topic 3: What have been the outcomes of the decision for the patient and family?

1. In what way(s) has the COVID-19 public health and economic crisis impacted your ability to care for X?
2. Has X's wellbeing changed due to the impact of the coronavirus?
3. What is the most useful service X is getting right now?
4. What would you like to see more of in terms of services?

Close-out

Thank participant for participating; emphasize how helpful s/he's been. Make sure s/he knows how to contact you if he has any questions or concerns.
