# Supplementary material for: Caregiving for Older Adults With Dementia During the Time of COVID-19: A Multi-State Exploratory Qualitative Study
Source: J Appl Gerontol. 2023 May 26;42(10):2078–88. doi: 10.1177/07334648231175414 (PMC10214026; doi:10.1177/07334648231175414)
Supplement: Supplemental Material - Caregiving for Older Adults With Dementia During the Time of COVID-19: A Multi-State Exploratory Qualitative Study [file sj-pdf-3-jag-10.1177_07334648231175414.pdf]

| Appendix C: Interview Codebook | |
|---|---|
| **Topic 1: Caregiver Decision Making** | |
| **Code Name** | **Code Description and Questions** |
| Care decision conflicts | Has there been challenges or conflicts with making care decisions for x? |
| Care decision employment and schedules | How were care decisions effected by the employment and work schedules of those caring for x? |
| Care decision physical safety | How were care decisions effected by the consideration of physical safety for x? |
| Care decision website | Did the respondent use websites to aid in making care decisions for x? |
| Care decision-family helpers | Have family members of x besides the respondent helped with any care decisions? |
| Care decision-medical or physician helpers | Have medical professionals helped with any care decisions regarding x? |
| Care decision-other social network helpers | Have people in the respondent and x's social network including friends, neighbors, church community, etc. helped with any care decisions regarding x? |
| Care decision-social work helpers | Have social work professionals helped with any care decisions regarding x? |
| Care options available or eligible for | Care options that are available to x that they are eligible for |
| Caregiver support groups online or in-person | Has the respondent participated in caregiving support groups? |
| COVID-19 and decision making | Discussion of decision making within the context of COVID-19 |
| Evaluation of quality of care | How did the respondent evaluate the quality of care when making care decisions for x? How did the evaluation of quality-of-care effect care decisions for x |
| HCBS consideration | Has the respondent considered home and community-based care options such as a home health aide, meals on wheels, home nursing care, etc.? |
| Nursing home consideration | Has the respondent considered nursing care for x? |

| | |
|---|---|
| Other care options considered | Has the respondent considered other care options such as adult day care, senior activities, church activities, etc.? |
| Tipping point | Discussion of an event that caused the change in type or extent of care |
| **Topic 2: State Policy, Insurance, Finances, and Other Service Coverage** | |
| **Code Name** | **Code Description and Questions** |
| Applying for insurance or other services | Respondents' experiences applying for insurance and other services for x |
| Caregiver being paid for work | Does the respondent receive compensation for caring for x? |
| Caregiving as unpaid labor | Discussion of the caregiving of x as unpaid labor |
| Confusion about insurance | Did the respondent experience confusion regarding insurance including coverage, eligibility, application process, etc.? |
| COVID-19 and state policy | Discussion of policies within the context of COVID-19 |
| Financial burden | Discussion of the financial burden of caring for x |
| Help with applying for insurance or other services | Did respondent or x receive help applying for insurance or other services? |
| Insurance challenges | Discussion of challenges with insurance |
| Insurance successes | Discussion of successes with insurance |
| Other financial contributions | Financial help received from entities outside of insurance and other formal services such as cash assistance from family, charity from church, GoFundMe, etc. |
| Other service coverage (ex. "Covered by the state") | Did the respondent discuss other types of care coverage such as services "covered by the state," community or charity services, etc.? |
| Services paid of out of pocket | Discussion of services paid out of pocket |
| **Topic 3: Caregiving Experiences** | |
| **Code Name** | **Code Description and Questions** |
| Advice for other caregivers | Respondents' advice for other caregivers |
| Caregiver challenges | Challenges experienced by respondent while caring for x |

| | |
|---|---|
| Caregiving successes | Successes experienced by respondent while caring for x |
| COVID-19 and caregiver experiences | Respondents' experiences with COVID-19 |
| Desired services | Services that are desired by the respondent that x is not receiving |
| family and social network help with care | The respondent's experiences with family and other social network help with the care of x |
| Future care considerations | Respondents' discussion of possible future care changes |
| Home modifications and safety | Home modifications that the respondent has made while caring for x |
| No outside help with care | Respondents' experiences of receiving no outside help with the care of x |
| Personal and emotional burdens and caregiver stress | Respondents' experiences with personal and emotional burdens related to caregiving and overall caregiver stress |
| Service gaps or waitlists | Respondents' experiences with having to wait for services or experiences gaps in services for x |
| Tasks performed (typical day question) | The general tasks the respondent performs while caring for x |
| Useful services | Respondents' experiences with useful services for x |
| **Topic 4: Experiences with Formal Care** | |
| **Code Name** | **Code Description and Questions** |
| Adult day care experiences | Respondents' discussion of experiences with adult day care services |
| Challenges with services received | Respondents' discussion of the challenges experienced with the formal services received |
| COVID-19 and formal care | Discussion of formal care within the context of COVID-19 |
| Home care experiences | Respondents' discussion of home care experiences |
| Medical professional experiences | Respondents' discussion of experiences with medical professionals |
| Nursing home experiences | Respondents' discussion of nursing home experiences |
| Social worker or case manager experiences | Respondents' discussion of experiences with social workers or case managers |

| | |
|---|---|
| Successes with services received | Respondents' discussion of the successes experienced with the formal services received |
| Support group experiences | Respondents' discussion of experiences with caregiver support groups |
| **Topic 5: Background** | |
| **Code Name** | **Code Description and Questions** |
| Demographics | Discussion of respondent and x's age, gender, race, and income |
| Duration of caregiving | How long has the respondent been a caregiver to x? |
| Level of care needed | How much care does x need (ex. driving to apts., bathroom care, medication management, etc.)? |
| Relationship between caregiver and x | Discussion of the relationship between caregiver and x |
| Residency change | Has the respondent or x changed residency since x has needed care? |
